# The role of TCF-1^+^CD8^+^ exhausted progenitors and TCF-1 in graft-versus-host responses

**DOI:** 10.1172/jci.insight.181568

**Published:** 2025-10-08

**Authors:** Kevin Quann, Faruk Sacirbegovic, Sarah Rosenberger, Emily R. McFerran, Kentin C. Codispot, Laura Garcia-Dieguez, Alexander M. Rowe, Wenzhong Wei, Dhanpat Jain, Jennifer M. McNiff, Warren Shlomchik

**Affiliations:** 1 Department of Medicine, Division of Malignant Hematology and Medical Oncology, University of Pittsburgh, Pittsburgh, Pennsylvania, USA.; 2 Starzl Transplantation Institute, University of Pittsburgh/University of Pittsburgh Medical Center, Pittsburgh, Pennsylvania, USA.; 3 Department of Immunology, University of Pittsburgh, Pittsburgh, Pennsylvania, USA.; 4 Department of Pathology and; 5 Department of Dermatology, Yale University School of Medicine, New Haven, Connecticut, USA.

**Keywords:** Immunology, Transplantation, Adaptive immunity, Bone marrow transplantation, T cells

## Abstract

In allogeneic hematopoietic transplantation, donor αβ T cells attack recipient tissues, causing graft versus host disease (GVHD). A longstanding question has been how GVHD is maintained despite T cell exhaustion from chronic alloantigen stimulation. In other exhaustion models, CD8 responses are sustained by CD39^lo^Tim-3^lo^Tox^hi^TCF-1^hi^ precursor exhausted T cells (T_PEX_). Here we characterize CD8^+^ T_PEX_ in the B6(H-2^b^)→129(H-2^b^) GVHD model wherein responses against the minor histocompatibility antigen H60 can be tracked using MHCI-tetramers (Tet^H60^). Early after transplant, Tet^H60+^ CD8 cells were uniformly PD-1^hi^Tox^hi^, whereas Tet^H60–^ cells also had PD-1^lo^Tox^lo^ cells, indicative of more diverse antigen experiences. Among Tet^H60+^ and Tet^H60–^ populations were CD39^lo^TCF-1^hi^ cells. Upon competitive retransplantation, Tet^H60+^CD39^lo^TCF-1^hi^ cells outcompeted Tet^H60+^CD39^hi^TCF-1^lo^ cells and underwent self-renewal, whereas CD39^hi^TCF-1^lo^ cells did not yield TCF-1^hi^ cells. To test the role of TCF-1, we studied CD8 cells lacking long TCF-1 isoforms (*p45^–/–^*). *P45^–/–^* cells were outcompeted by WT cells when transplanted into 129 recipients, though they expanded similarly in syngeneic recipients. In the B6→C3H.SW(H-2^b^) model, *p45^–/–^* CD8 cells caused less weight loss than did WT CD8 cells; however, histopathologic GVHD was similar in both groups. *P45^–/–^* and WT CD8 cells also had similar graft versus leukemia activity. These results highlight the complex biology of TCF-1 in supporting alloreactive T cell function.

## Abstract 2

Donor-derived progenitor-like CD8^+^ T cells expressing TCF-1 develop early post-transplant and sustain effector T cell outgrowth in graft-vs-host disease target tissues.

## Graphical abstract

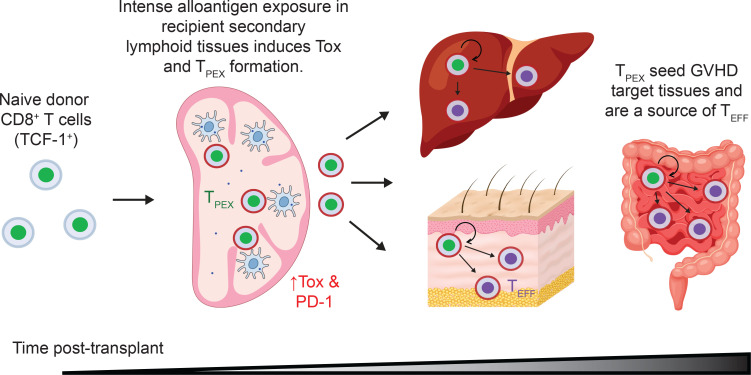

## Introduction

Allogeneic stem cell transplant (alloSCT) is a potentially curative therapy for patients with hematological malignancies and acquired or inherited disorders of hematopoiesis. Alloreactive donor αβ T cells in the graft that recognize the recipient as “non-self” promote engraftment by attacking host immune and other hematopoietic cells, and in the application of alloSCT for hematologic malignancies, they can target neoplastic cells, thereby mediating the graft versus leukemia (GVL) effect. However, alloreactive donor T cells can also target normal host tissues, causing graft versus host disease (GVHD) (1). Because of GVHD, all recipients of T cell–replete grafts receive immunosuppressive agents. Nonetheless, GVHD and the consequences of immunosuppression remain major causes of post-alloSCT morbidity and mortality, which limit the more widespread adoption of alloSCT in treatment of both malignant and nonmalignant conditions (1, 2). Therefore, understanding the mechanisms that underly the initiation and maintenance of GVHD by alloreactive T cells is a necessary step toward the development of more effective strategies to mitigate it.

In major histocompatibility complex–matched (MHC-matched) alloSCT, which are the majority of those performed clinically, donor T cells recognize minor histocompatibility antigens (miHAs), which are the peptide products of genetic polymorphisms that distinguish recipients from donors (3, 4). The early priming of alloreactive CD8^+^ T cells in GVH responses is driven by host antigen presenting cells (APCs) that survive the conditioning regimen and that directly and efficiently present miHAs (5). Subsequently, alloreactive CD8 cells may also see their cognate miHAs directly presented by recipient nonhematopoietic tissues or by donor-derived DCs that cross-present miHAs derived from nonhematopoietic cells, which are an unlimited source of antigen. This efficient and sustained antigen presentation can result in exhaustion of alloreactive T cells, such that they share phenotypic, functional, and metabolic features of exhausted T cells characterized in chronic viral infections and in antitumor responses (6–11).

Although CD8 cells in these viral and tumor models have been described as being globally exhausted, a subpopulation of cells emerges early in the response that retains the ability to proliferate when rechallenged with antigen, thereby generating new effectors, and these are the cells that are “reinvigorated” by reagents that target PD-1 and PD-L1. Such T cells have been named precursor exhausted T cells (T_PEX_) and canonically express the transcription factor TCF-1 (encoded by *Tcf7*) (12–17), which is required for normal T cell thymic maturation (18, 19). T_PEX_ formation is dependent upon the transcription factor thymocyte selection-associated high mobility group box protein (Tox),_,_ which is upregulated early after initial T cell activation in settings of chronic antigen availability and is expressed by canonical T_PEX_ (20–24).

We recently described the formation of alloreactive T_PEX_-like CD4 and CD8 cells in several mouse models of GVHD (11, 25), a situation wherein there is strong and sustained antigen stimulation of T cells. In systems wherein CD4^+^ T cell receptor–transgenic T cells target a model miHA expressed ubiquitously in MHC-matched recipients, or in which polyclonal donor CD4^+^ T cells mediate GVHD in MHC-mismatched recipients, Tox^hi^ cells form in secondary lymphoid tissues (SLT) early after transplant, coincident with the development of TCF-1^hi^CD39^lo^ T_PEX_. These T_PEX_ distribute to other tissues, including those canonically affected by GVHD. Parabiosis experiments, analysis of T cell clone tissue distributions, and adoptive transfer experiments wherein TCF-1^hi^CD39^lo^ CD4 cells outcompeted TCF-1^lo^CD39^hi^ CD4 cells (both harvested from GVHD mice) in new transplant recipients further suggested that these tissue-resident CD39^lo^TCF-1^+^ T_PEX_ are a critical source of effectors that maintain GVHD locally within affected tissues (25).

In the C3H.SW (H-2^b^; H60^–^)→B6.H60 (H-2^b^; H60^+^) CD8^+^ T cell GVHD model, we also observed the early emergence of splenic donor PD-1^hi^Tox^hi^ CD8 cells, including those reactive against the immunodominant miHA H60 (detected by a tetramer against H60; Tet^H60+^). A fraction of these Tet^H60+^ and Tet^H60–^ PD-1^hi^Tox^hi^ cells expressed TCF-1 and were also Tim-3^lo^CD39^lo^Ly108^hi^, consistent with their being T_PEX_. However, we did not perform functional studies on these putative T_PEX_ and only studied spleen and bone marrow (BM) (11). Furthermore, in no GVHD model have we specifically assessed the importance of TCF-1 activity. Here, we utilize the MHC-matched B6 (H-2^b^; H60^–^)→129 (H-2^b^; H60^+^) model, wherein we can track H60-specific CD8^+^ cells. We also use donor CD8 cells lacking the long β-catenin-binding isoform of TCF-1 (*p45^–/–^*) to study the functional importance of TCF-1 in CD8^+^ T cell GVHD responses.

## Results

### TCF-1^+^ T_PEX_ develop and persist in GVHD target tissues.

**Figure 1:**
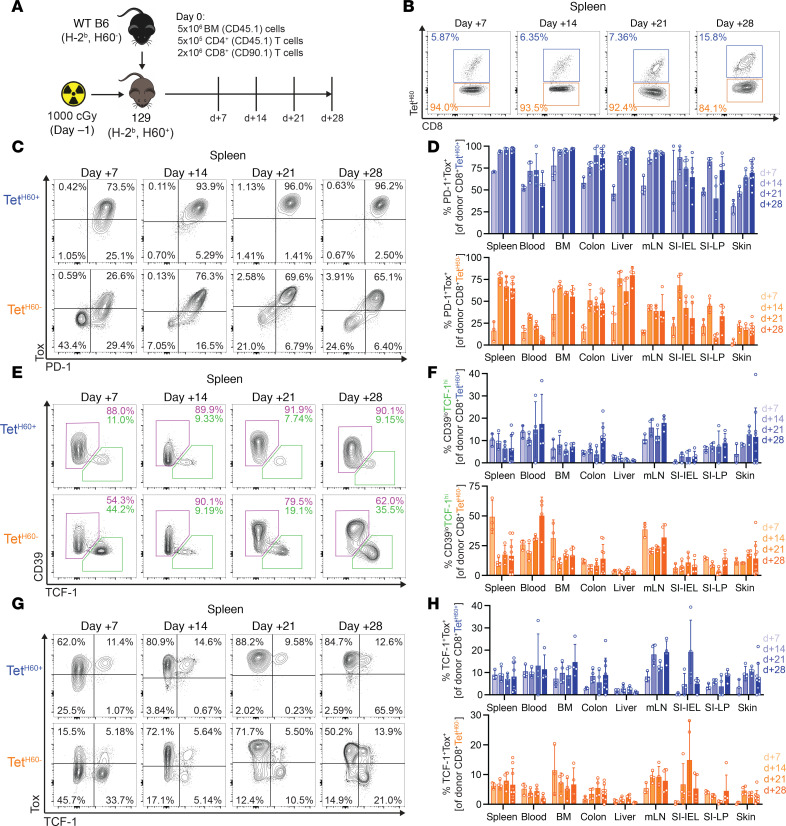
Time-course of TCF-1^+^ T_PEX_ development and T_PEX_ immunophenotypes. (**A**) Experimental design. In total, 129 recipients were lethally irradiated and reconstituted with BM and CD4^+^ T cells from B6 CD45.1^+^ donors and CD8^+^ T cells from CD90.1^+^ donors. Cohorts of mice (*n* = 3–9 each across 2 experiments) were sacrificed at days +7, +14, +21, and +28 after transplant for analysis of T cells in secondary lymphoid tissues and in GVHD target tissues. (**B**) Representative flow plot with Tet^H60^ demonstrating discreet populations of Tet^H60+^ and Tet^H60–^ cells. (**C** and **D**) Tox and PD-1 expression of Tet^H60+^ and Tet^H60–^ CD8 cells. Representative staining (**C**) and quantitation in tissues (**D**; mLN, mesenteric lymph node; SI-IEL, small intestine intraepithelial lymphocytes; SI-LP, small intestine lamina propria). (**E** and **F**) Development of Tet^H60+^ and Tet^H60–^ CD39^lo^TCF-1^hi^CD8^+^ cells over time. Representative staining (**E**) and quantitation in tissues (**F**). (**G** and **H**) Tet^H60+^ TCF-1^hi^ cells are Tox^hi^, whereas there are Tox^lo^ and Tox^hi^ TCF-1^hi^ cells among Tet^H60–^ cells. Representative staining (**G**); quantitation in **H**.

To study the generation and function of alloreactive CD8^+^ T_PEX_ in GVHD responses, we employed the B6→129 GVHD model. An advantage of this system is that 129 mice (but not B6 mice) express the immunodominant miHA H60, allowing the tracking of H60-reactive T cells with MHCI-tetramers (Tet^H60^) (Supplemental Figure 1A; supplemental material available online with this article; https://doi.org/10.1172/jci.insight.181568DS1) (11, 26–28). A second benefit is that most gene-modified mice are available on the B6 background, which enables mechanistic studies. We first investigated the time-course of the development of T_PEX_-like cells. Lethally irradiated 129 recipients were reconstituted with B6 CD45.1 BM and CD4^+^ T cells and B6 CD90.1 CD8^+^ T cells. Cohorts were sacrificed weekly through day +28 and infiltrating T cells in SLTs, and GVHD target tissues were analyzed (Figure 1A, Supplemental Figure 1B, and Supplemental Figure 1C).

At day +7 in spleen and mesenteric lymph node (mLN), sites of T cell priming (29, 30), clear Tet^H60+^ and Tet^H60–^ populations were already present (Figure 1B and Supplemental Figure 1D). Most Tet^H60+^ cells were PD-1^hi^Tox^hi^ and a majority of these had also upregulated Tim-3 (Figure 1, C and D, and Supplemental Figure 1, E and F). In contrast, among Tet^H60–^ cells there were distinct populations of PD-1^hi^Tox^hi^ and PD-1^lo^Tox^lo^ cells with a substantial fraction of PD-1^hi^ cells also expressing Tim-3 (Figure 1, C and D, and Supplemental Figure 1, E and F). Whereas the Tet^H60–^PD-1^hi^Tox^hi^ cells likely had an antigen experience similar to that of Tet^H60+^ cells, Tet^H60–^PD-1^lo^Tox^lo^ cells may have had less antigen stimulation and could also include cells that did not respond to miHAs but underwent lymphopenia-induced proliferation (31–33). Among both Tet^H60+^ and Tet^H60–^ cells were CD39^lo^TCF-1^hi^Tim3^lo^ cells (Figure 1, E and F, and Supplemental Figure 1G). As Tet^H60+^ cells were nearly all PD-1^hi^Tox^hi^, so too were the CD39^lo^TCF-1^hi^ cells, suggesting that T_PEX_ development had already begun (Figure 1, G and H). Among the Tet^H60–^ cells there were also TCF-1^hi^Tox^hi^ T_PEX_-like cells (Figure 1, G and H); however, unlike in the Tet^H60+^ population, there were also TCF-1^hi^ cells that were Tox^lo^ (Figure 1, G and H). Of note, within this Tet^H60–^ Tox^lo^ TCF-1^hi^ population were CD62L^+^ cells, both CD44^hi^ and CD44^lo^, whereas Tet^H60–^TCF-1^hi^ Tox^hi^ cells were mostly CD62L^–^ beyond day 7, as were all TCF-1^hi^ Tet^H60+^ cells (Supplemental Figure 1H).

At day +14 in spleen, most Tet^H60+^ cells remained PD-1^hi^Tox^hi^. However, fewer Tet^H60–^ cells were PD-1^lo^Tox^lo^ than was observed at day +7, potentially indicative of the progressive expansion of CD8 cells targeting well-presented miHAs other than H60 (Figure 1, C and D). Among splenic Tet^H60+^ and Tet^H60–^ cells, 9.7% (±3.4% SD) and 11.3% (±3.6% SD) were CD39^lo^TCF-1^hi^, respectively (Figure 1, E and F). The day +21 and +28 data were similar to those at day +14 (Figure 1, C–F).

Tet^H60+^ and Tet^H60–^ CD8 cells also distributed to nonlymphoid tissues, including those canonically affected by GVHD (Supplemental Figure 1D). Except for liver, alloreactive CD8 cells isolated from tissues were largely excluded from intravenous CD45.2 labeling, indicative of intraparenchymal distributions (Supplemental Figure 1I). The phenotypes of Tet^H60+^ cells were consistent across mice and each tissue at each time point; most were PD-1^hi^Tox^hi^ with small populations of CD39^lo^TCF-1^hi^ cells (Figure 1, D and F, and Supplemental Figures 2 and 3). Among Tet^H60–^ cells, there were again distinct populations of PD-1^hi^Tox^hi^ and PD-1^lo^Tox^lo^ cells (Figure 1D and Supplemental Figure 3), with TCF-1^hi^ cells found in both Tox^hi^ and Tox^lo^ subsets (Figure 1H and Supplemental Figure 4). However, there was variability in these fractions across tissues.

### TCF-1^+^ T_PEX_ have increased potential for expansion in GVHD.

**Figure 2:**
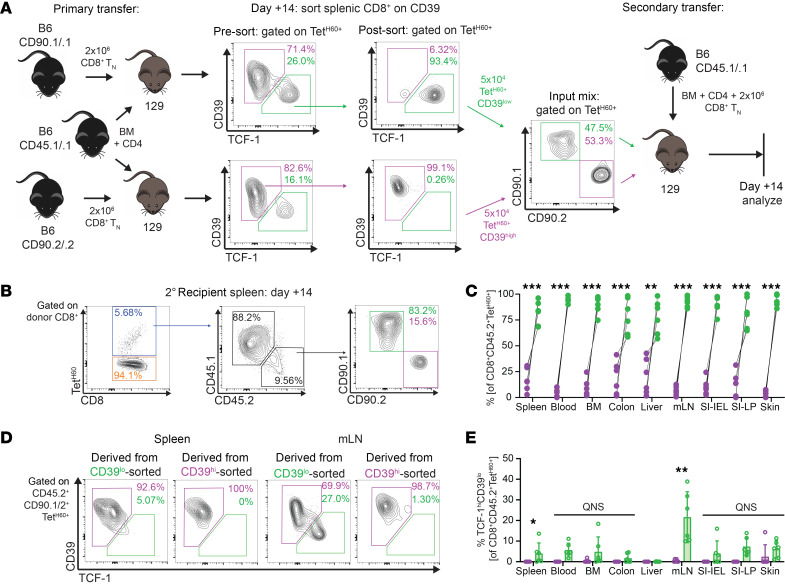
CD39^lo^TCF-1–enriched CD8 cells outcompete CD39^hi^TCF-1–depleted CD8 cells on retransplantation. (**A**) Competitive secondary transfer experimental design. In parallel, primary 129 recipients were lethally irradiated and reconstituted with B6 CD45.1 BM and CD4^+^ cells and CD90.1^+^ or CD90.2^+^ B6 CD8 T cells. At day +14, CD8^+^ donor T cells from spleens were sorted into CD39^hi^ and CD39^lo^ fractions. New unmanipulated 129 recipients were lethally irradiated and reconstituted with B6 CD45.1 BM, CD4, and CD8 cells, and congenically distinct CD39^hi^ and CD39^lo^ CD8 cells containing the same number of Tet^H60+^ cells. Shown are pre- and post-sort analyses (right panels) from 1 of 2 replicated experiments. (**B** and **C**) At day +14 after the second transplant, the progeny of congenic CD39^hi^ and CD39^lo^ sorted Tet^H60+^ cells were enumerated. Representative flow cytometry (**B**) and quantitation (**C**). **P < 0.01, ***P < 0.001 by 2-sided Student’s *t* test. (**D** and **E**) Representative TCF-1 and CD39 staining of the progeny of transferred CD39^hi^ and CD39^lo^ sorted cells 14 days after secondary transfer and their quantitation in tissues (QNS, quantity of CD39^hi^-sorted cells not sufficient for statistical comparison). *P <0.05, **P < 0.001 by 1-sided Student’s *t* test.

To test the fitness of TCF-1^hi^ T_PEX_, we competed them against TCF-1^lo^ effectors (T_EFF_) using a secondary transfer approach. We first created congenically distinct alloreactive CD8^+^ T cells by transplanting 129 mice with B6 CD45.1 BM and either CD45.2 B6 CD90.1^+^ or CD90.2^+^ CD8^+^ T cells. At day +14, donor-derived splenic CD8 cells were sorted into CD39^lo^ and CD39^hi^ subsets to create populations that were enriched for (CD39^lo^) or depleted of (CD39^hi^) TCF-1^hi^ cells as confirmed by a postsort analysis (Figure 2A). We chose to compete T cells harvested at day 14, as the T_PEX_ population appeared to be stable at this point and therefore unlikely to be contaminated by effectors that had yet to downregulate TCF-1. To minimize variation introduced by T cells having a heterogeneous mix of target antigens, we focused only on H60-reactive CD8 cells. Sorted congenic CD39^hi^TCF-1^lo^ and CD39^lo^TCF-1^hi^ containing equal numbers of Tet^H60+^ cells were transferred along with CD45.1 B6 BM and unmanipulated B6 splenic CD8 cells into a second cohort of irradiated 129 mice (Figure 2A). The frequencies and phenotypes of the Tet^H60+^ progeny of CD39^hi^ and CD39^lo^ cells were analyzed 14 days later.

In all tissues, Tet^H60+^ progeny of CD39^lo^TCF-1^hi^ cells outnumbered Tet^H60+^ progeny of CD39^hi^TCF-1^lo^ cells by an average of 21-fold (Figure 2, B and C). Most progeny of CD39^lo^TCF-1^hi^ cells became CD39^hi^TCF-1^lo^ T_EFF_ (Figure 2, D and E), consistent with what we reported for CD4 cells in GVHD (25) and which has been described for CD8^+^ T_PEX_ in LCMV clone 13 infection when similar populations were studied through adoptive transfer approaches (13, 15, 34). Importantly, in most tissues in most mice, there were progeny of CD39^lo^ cells that retained TCF-1 expression, which could represent a self-renewing population of T cells with preserved stemness. In contrast, there were few progeny of CD39^hi^ cells expressing TCF-1. We only recovered sufficient progeny of CD39^hi^ cells from spleen, mLN, and liver to allow for a statistical comparison of the frequencies of TCF-1^hi^ cells. In spleen and mLN higher percentages of sorted CD39^lo^ progeny were TCF-1^hi^ (Figure 2E). Taken together, these data suggest that TCF-1^hi^ cells can self-renew, whereas CD39^hi^ TCF-1^lo^ cells are largely unable to differentiate into TCF-1^hi^ cells.

### Full-length TCF-1 is required for optimal expansion of alloreactive CD8 cells in GVHD responses.

**Figure 3:**
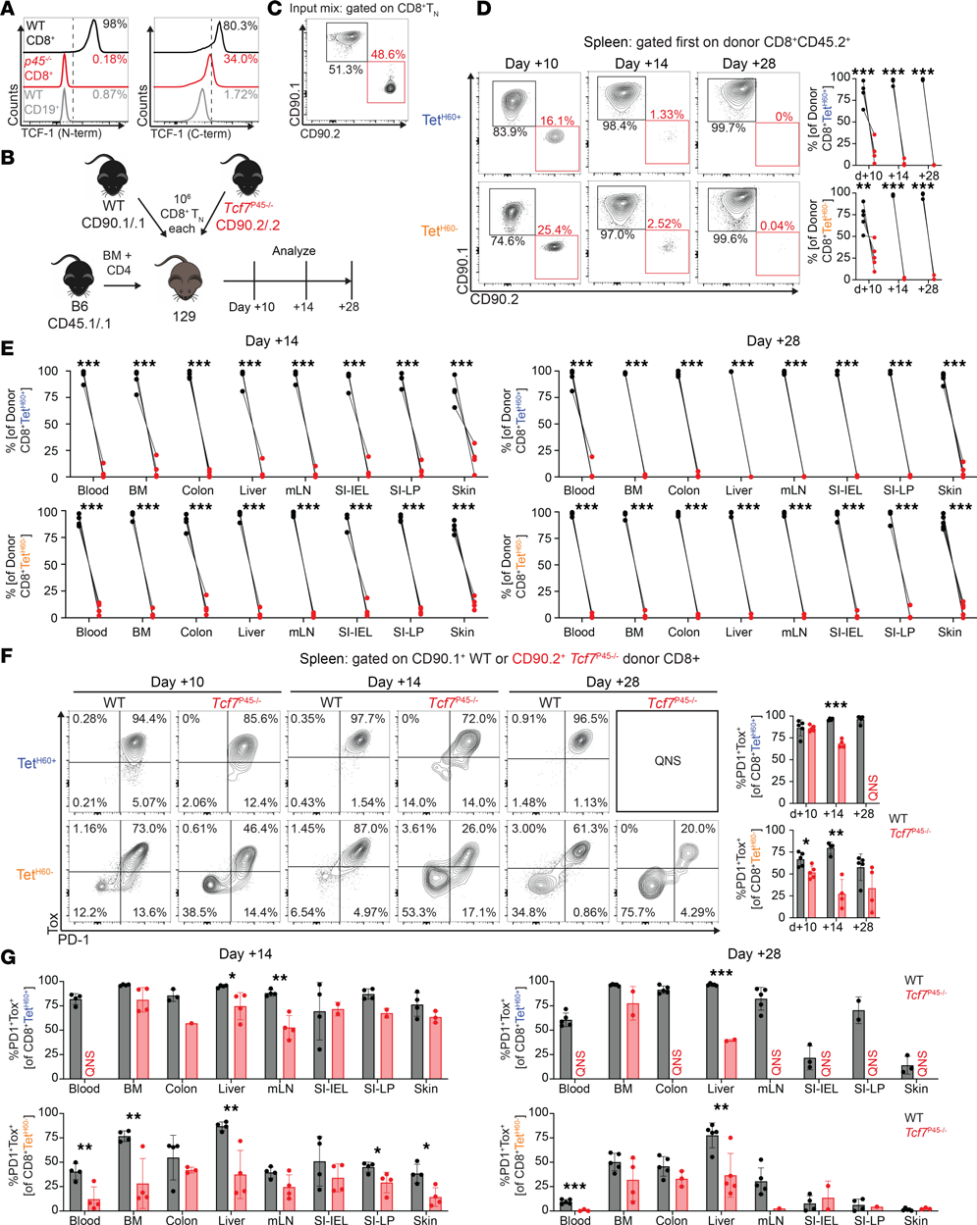
*P45^–/–^* CD8 cells are outcompeted by WT CD8 cells. (**A**) Staining for TCF-1 using N-terminal and C-terminal antibodies confirms the lack of the N-terminal β-catenin binding domain in *p45^–/–^* CD8^+^ donor cells (red), similar to negative control CD19^+^ B cells (gray). TCF-1 C-terminal inclusive isoforms are still detected in *p45^–/–^* CD8 cells using a C-terminal antibody, though expression is lower than in WT cells (black). (**B**) Experimental design. WT CD90.1^+^ CD8 cells were transferred in a 1:1 ratio with CD90.2^+^ *p45^–/–^* cells into lethally irradiated 129 recipients with B6 CD45.1 BM and CD4 cells. Tissues were harvested from recipients on days +10 (spleen only), +14, and +28. (**C**) Flow cytometry of the T cell mix prior to infusion, gated on CD8^+^CD44^–^CD62L^+^ cells. (**D**) Representative flow plots from 2 separate experiments (left panel) and quantitation of Tet^H60+^ and Tet^H60–^ progeny of competed donor CD8 cells in spleen at days +10, +14, and +28. (**E**) Quantitation of Tet^H60+^ and Tet^H60–^ progeny of competed CD8 cells at days +14 and +28 in other tissues and blood. (**F**) Representative Tox and PD-1 staining (left panel) and quantitation (right panel) of PD-1^+^Tox^+^ progeny of *p45^–/–^* and WT cells in spleen over time. (**G**) Quantitation of PD-1^+^Tox^+^ Tet^H60+^ and Tet^H60–^ progeny of *p45^–/–^* and WT CD8 cells in tissues at days +14 and +28. QNS, quantity of *p45^–/–^* cells not sufficient for statistical comparison. *P < 0.05, **P < 0.01, ***P < 0.001 by 2-sided Student’s *t* test.

In the time-course analysis (Figure 1), we observed T_PEX_ development, manifest by upregulation of Tox, PD-1, and continued expression of TCF-1, as early as day +7 among both Tet^H60+^ and Tet^H60–^ splenic CD8 cells. If these SLT-resident T_PEX_, which also distribute to nonlymphoid tissues, are important for inducing and maintaining GVHD, and if TCF-1 itself is important for T_PEX_ function, a prediction would be that TCF-1–deficient CD8 cells from unmanipulated donors should have impaired expansion in GVHD models. Constitutive *Tcf7*-KO mice have a block in the development of early thymic progenitors and double-negative thymocytes and, therefore, would not have been appropriate for such studies (18, 35). We therefore chose as a source of *Tcf7*-impaired T cells mice that lack the full-length (p45) isoform of TCF-1, wherein there is a deletion of the N-terminal portion of TCF-1 that binds β-catenin. Such mice were created by knocking into the first intron of *Tcf7* an EGFP expression cassette engineered with a 5′ splice acceptor and a 3′ polyadenylation signal such that exon 1 splices to the cassette without incorporating subsequent exons (*p45^–/–^* mice) (36, 37). However, transcription can be initiated at an alternate transcription start site upstream of the third exon, enabling expression of the short p33 and p30 isoforms lacking the N-terminal β-catenin binding domain. These mice do not have a stage-specific block in thymocyte development, though the number of thymocytes is modestly reduced (37). Staining with an N-terminal-specific TCF-1 antibody confirmed the absence of full-length TCF-1 from *p45^–/–^* mice, whereas staining with a C-terminal-specific antibody, which captures both short- and long TCF-1 isoforms, demonstrates preserved expression of p33 in CD8 cells from *p45^–/–^* donors as described previously (37) (Figure 3A).

Irradiated 129 recipients were reconstituted with B6 CD45.1 BM and a 1:1 mix of CD8 cells containing 1 × 10^6^ WT CD90.1^+^ and 10^6^ *p45^–/–^* CD90.2^+^ naive T cells (T_N_) cells (experimental design, Figure 3B, flow cytometry confirmation of the WT and *p45^–/–^* T cell mix, and Figure 3C). We initially focused on early T cell expansion in spleen and analyzed cells at days +10 or +14 in independent experiments. In spleen, WT T cells greatly outperformed *p45^–/–^* cells among both Tet^H60+^ and Tet^H60–^ subsets (Figure 3D). The reduced competitiveness of *p45^–/–^* CD8 cells in spleen was paralleled in other tissues analyzed at days +14 and +28, wherein the majority of Tet^H60+^ and Tet^H60–^ CD8 cells were WT (Figure 3E). Despite impaired expansion, splenic *p45^–/–^* Tet^H60+^ cells were mostly PD-1^hi^Tox^hi^ (Figure 3F). Among Tet^H60–^ cells, a smaller percentage of *p45^–/–^* progeny were PD-1^hi^Tox^hi^, suggesting that there could have been selective culling of cells that had the most sustained TCR stimulation (Figure 3F). However, the decrease in the percentage of PD-1^hi^Tox^hi^ cells cannot alone account for the reduced number of Tet^H60–^ *p45^–/–^* progeny, indicating that the long isoform of TCF-1 was also required for optimal expansion of cells that were not induced to express high levels of Tox and PD-1. These features of PD-1 and Tox expression in splenocytes were also observed in donor CD8 cells infiltrating other tissues (Figure 3G). Taken together, these data demonstrate that GVH-reactive CD8 cells, and especially those activated sufficiently to induce high levels of Tox and PD-1, require the long TCF-1 isoforms for optimal expansion.

### Full-length TCF-1 is not required for optimal expansion of CD8 cells with less intense antigen challenge.

**Figure 4:**
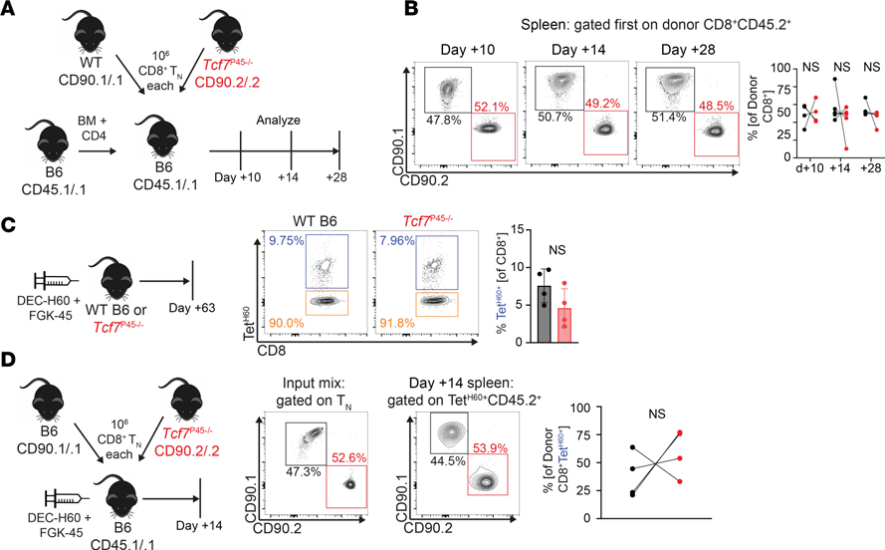
*P45^–/–^* CD8 cells are not impaired in lymphopenia-induced proliferation or response to vaccination. (**A**) WT CD90.1^+^ CD8 cells were transferred in a 1:1 ratio with CD90.2^+^ *p45^–/–^* CD8 cells, along with CD45.1 BM and CD4 cells into lethally irradiated syngeneic B6 CD45.1 recipients. At days +10, +14, and +28, spleens were harvested to enumerate frequencies of donor CD8 cells. (**B**) Representative staining from 2 separate experiments and quantitation. (**C**) WT and *p45^–/–^* mice were vaccinated against H60 with DEC-H60 and an anti-CD40 agonist antibody (FGK45; left panel). Two months later, spleens were enumerated for the presence of Tet^H60+^ CD8 cells. Representative flow (middle panel) and quantitation (right panel) are shown. (**E**) In total, 1 × 10^6^ CD8^+^ T_N_ from CD90.1 WT and CD90.2 *p45^–/–^* spleens were cotransferred into WT CD45.1 B6 mice, followed by vaccination with DEC-H60 and FGK45. Two-weeks later, competing donor cells were enumerated from spleens (left panel). Representative staining (middle panel) and quantitation (right panel) are shown. NS: not significant by 2-sided Student’s *t* test.

We first tested whether long TCF-1 isoforms are required for optimal lymphopenia-induced proliferation, which is also dependent on TCR stimulation (32). In contrast to what was observed in 129 recipients, *p45^–/–^* CD8 cells were not at a competitive disadvantage when cotransplanted with WT CD8 cells into syngeneic B6 mice, indicating that the long TCF-1 isoform is not required for lymphopenia-induced proliferation (Figure 4, A and B).

We next tested whether long TCF-1 isoform is required for responses against H60 delivered by vaccination instead of in the context of alloSCT. In one approach, *p45^–/–^* and WT mice were immunized against H60 using an anti-DEC205 antibody modified to express the immunodominant LTFNYRNL epitope from H60, along with an agonist antibody against CD40 (clone FGK45) as an adjuvant (28). There were similar numbers of WT and *p45^–/–^* Tet^H60+^ CD8 cells in spleen after 8 weeks (Figure 4C). In a second approach, we cotransferred 1 × 10^6^ CD8 cells from CD45.2^+^CD90.1^+^ WT and CD45.2^+^CD90.2^+^ *p45^–/–^* mice into CD45.1^+^ B6 mice followed by DEC-H60/FGK45 immunization. Mice were sacrificed 14 days later to enumerate Tet^H60+^ CD8 cells from each T cell source. Although there was mouse to mouse variability, possibly due to stochastic effects operating on small numbers of anti-H60 precursors, overall, WT and *p45^–/–^* cells responded similarly (Figure 4D). Taken together, these results indicate that *p45^–/–^* T cells are not impaired in responding to H60 as presented after immunization, despite their competitive disadvantage when challenged against H60 in a GVHD model.

### P45^–/–^ CD8 cells cause less clinical but similar histopathologic GVHD.

**Figure 5:**
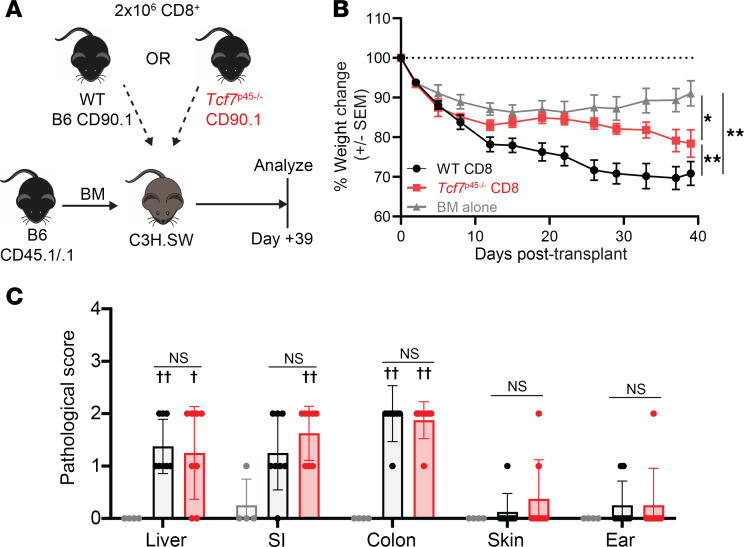
*p45^–/–^* cells cause less clinical but similar histologic GVHD. (**A**) Experimental design. C3H.SW recipients (H-2^b^) were irradiated with 1,000 cGy and reconstituted with 5 × 10^6^ T cell–depleted BM cells from B6 CD45.1^+^ mice with or without 2 × 10^6^ CD8^+^ T cells from WT B6 CD90.1^+^ or *p45^–/–^* CD90.1^+^ mice. (**B**) Recipients were followed for weight loss over time. *P < 0.05, **P < 0.001 by 1-way ANOVA. (**C**) Histopathological scores of recipient tissues. NS, not significant comparing WT to *p45^–/–^* by 2-sided Mann-Whitney *U* test. ^†^*P* < 0.05 and ^††^*P* < 0.01 comparing WT or *p45^–/–^* to BM alone control by 2-sided Mann-Whitney *U* test.

While the B6→129 model enables the tracking and study of CD8^+^ T cell responses against H60, CD8 cells alone do not induce robust GVHD. Therefore, to test whether full length TCF-1 is required for maximal CD8-mediated GVHD, we switched to the B6→C3H.SW (H-2^b^) model. C3H.SW recipients were irradiated and transplanted with T cell–depleted B6 CD45.1^+^ BM and CD8^+^ T cells from WT B6 CD90.1^+^ or *p45^–/–^* CD90.1^+^ mice (Figure 5A). Recipients of WT CD8 cells had significantly greater weight loss than did BM-only controls and *p45^–/–^* CD8 recipients (Figure 5B). Mice were sacrificed on day +39 and tissues were harvested for GVHD scoring as described previously (38). Contrary to the difference in weight loss, both WT and *p45^–/–^* CD8 cells caused similar degrees of histopathologic GVHD compared with BM-only controls in liver, small intestine, and colon, whereas skin was unaffected in this model (Figure 5C).

### P45^–/–^ CD8 cells mediate GVL.

**Figure 6:**
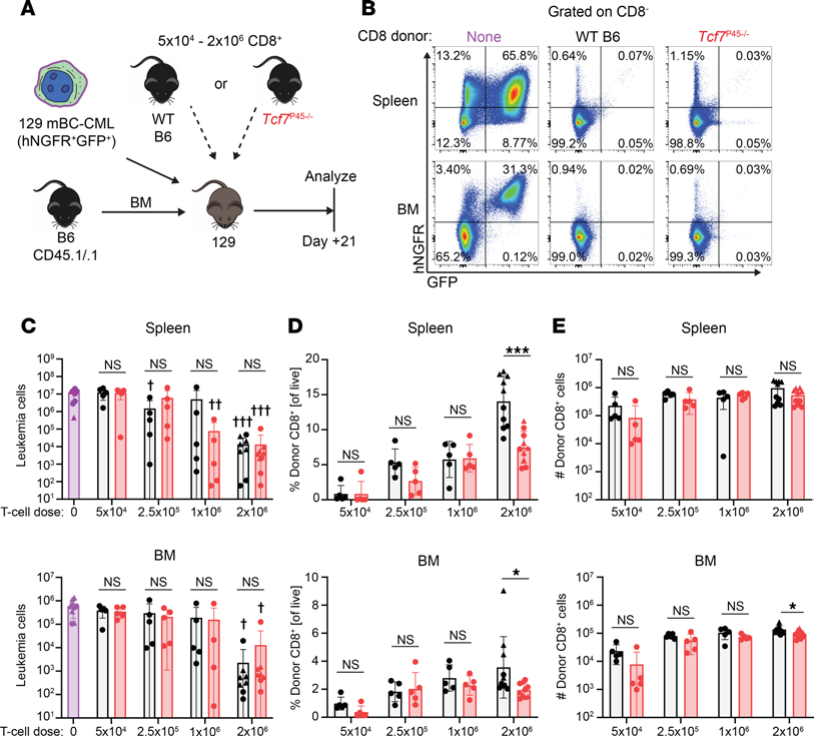
*P45^–/–^* CD8 cells mediate GVL. (**A**) Lethally irradiated 129 mice were reconstituted with B6 CD45.1 BM and 129-derived mBC-CML cells (containing 5 × 10^4^ that were CD11b^–^) with or without CD8 cells (5 × 10^4^ to 2 × 10^6^ per mouse) from B6 WT or *p45^–/–^* donors. Three-weeks later, GFP^+^humanNGFR^+^ mBC-CML cells (**B**, representative flow cytometry) were enumerated in spleen and BM (one femur; **C**). Data shown are combined from 2 separate experiments, represented by triangle and circle symbols. NS, not significant. *P < 0.05 and **P < 0.01 comparing recipients of WT or *p45^–/–^* CD8 cells; or ^†^*P* < 0.05, ^††^*P* < 0.01, or ^†††^*P* < 0.001 comparing a T cell recipient group to the no T cell group using a 2-sided Student’s *t* test.

To test the importance of full-length TCF-1 in GVL responses, we compared GVL mediated by *p45^–/–^* and WT CD8 cells against a mouse model of blast-crisis chronic myeloid leukemia (mBC-CML) in which the oncogenic fusion genes *bcr-abl* (linked to the expression of human NGFR) and *NUP98-HOXA9* (linked to GFP expression) are retrovirally transduced into BM cells 39, 40). Irradiated 129 mice were reconstituted with 129-derived mBC-CML cells and B6 BM with or without graded doses of WT or *p45^–/–^* CD8^+^ cells (Figure 6A, experimental design). Mice were sacrificed on day +21 to enumerate mBC-CML cells and donor T cells. In both spleen and BM, there were fewer mBC-CML cells in high-dose CD8^+^ T cell recipients (2 × 10^6^ / mouse) compared with mice that received lower CD8^+^ T cell doses (Figure 6, B and C). Surprisingly, mBC-CML numbers were similar in *p45^–/–^* and WT CD8 recipients at all doses. Consistent with what was observed in the competitive setting, at high CD8 cell doses (2 × 10^6^/mouse), we observed significantly fewer donor *p45^–/–^* donor CD8 cells as a percentage of total live cells compared with WT CD8 cells in spleen and BM (Figure 6D). However, there were no differences between percentages of *p45^–/–^* and WT cells at lower cell doses (Figure 6D), or when comparing total number of donor CD8 cells recovered at all dose levels (Figure 6E).

## Discussion

Here we report on the development and functionality of CD8^+^CD39^lo^TCF-1^hi^ T_PEX_ in GVHD, and on the role of TCF-1 itself in alloreactive CD8^+^ T cell responses. We employed a model wherein we could track CD8 cells responding to the well expressed immunodominant miHA H60.

Tet^H60+^ T_PEX_ developed early posttransplant in spleen and mLN, characterized by the coexpression of PD-1, Tox, and TCF-1. In contrast, among Tet^H60–^ cells, there were distinct populations of TCF-1^+^ cells that were PD-1^hi^Tox^hi^ or PD-1^lo^Tox^lo^, suggesting different pathways of development. Such TCF-1^hi^PD-1^lo^Tox^lo^ T cells could be responding to miHAs that are less well presented than H60 and therefore don’t induce Tox, leading to a differentiation pathway that may be more akin to T_CM_ development in acute LCMV models (41, 42). It is also possible that CD8 cells with lower avidity TCRs may not differentiate into T_PEX_, even if targeting well-presented miHAs. Another alternative is that PD-1^lo^Tox^lo^ cells may not be responding to miHAs at all and could be undergoing lymphopenia-induced proliferation driven by peptide/MHCI complexes shared between B6 and 129 mice. Distinguishing these possibilities will require determining the specificities of these TCF-1^hi^PD-1^lo^Tox^lo^ cells and characterizing the properties of their TCRs.

Activated donor T cells, both Tet^H60+^ and Tet^H60–^, distributed to nonlymphoid tissues, including those canonically affected by GVHD. Importantly, we found TCF-1^+^ CD8 cells in most tissues of most mice at all time points. In parallel to what was observed in spleen and mLN, among Tet^H60+^ cells, nearly all of those expressing TCF-1 had a classic CD39^lo^PD-1^hi^Tox^hi^ T_PEX_ phenotype, whereas tissue-resident Tet^H60–^ TCF-1^+^ cells were a mix of PD-1^hi^Tox^hi^ and PD-1^lo^Tox^lo^ cells.

To explore the functionality of T_PEX_, we performed competitive retransfer experiments wherein CD39^hi^ and CD39^lo^ CD8 cells harvested from spleens of mice at day +14 after BM transplantation (BMT) were infused into newly transplanted 129 mice. We focused on H60-reactive CD8 cells, as all Tet^H60+^ TCF-1^+^ cells had a classic T_PEX_ phenotype and were uniformly CD39^lo^. We were also concerned that there could be stochastic variability in responses to the unknown miHAs targeted by Tet^H60–^ cells that could have confounded data interpretation. Tet^H60+^ CD39^lo^ progeny greatly outperformed CD39^hi^ progeny in all tissues. In spleen and mLN, wherein there were sufficient progeny of each competed population to analyze, CD39^lo^ TCF-1^+^ cells could be found among CD39^lo^-sorted progeny. In contrast, such cells were rare if present at all among sorted CD39^hi^ progeny, suggesting that once CD39^hi^ cells are generated, they do not meaningfully differentiate into TCF-1^+^ cells, whereas CD39^lo^ cells are capable of self-renewal. Taken together, these results support a model wherein T_PEX_ are initially formed in SLT and then distributed to GVHD target tissues, where they form a durable source of alloreactive T_EFF_ (25).

Lee et al. recently described the competitive adoptive transfer of CD8 cells enriched for or depleted of TCF-1–expressing cells, harvested 7 days after transplant in an MHC-mismatched alloSCT model (43). They reported that the progeny enriched for TCF-1^+^ cells outcompeted cells depleted of TCF-expressing cells. While consistent with the present work and our previously published report on alloreactive CD4 cells (25), their design differed from ours in important ways. First, they transferred cells harvested only 7 days after transplant, making these likely to be contaminated by T cells that were only partially activated and not yet full-fledged T_PEX_. Second, only 16% of the PD-1^hi^Tim-3^lo^TCF-1–enriched cells actually expressed TCF-1, and progeny of TCF-1^lo^Tim-3^lo^ cells could have meaningfully contributed to the cells recovered. They also could not determine whether the Tim-3^hi^ and Tim-3^lo^ populations contained equally alloreactive repertoires, making it impossible to know whether the differences observed were due to repertoire or other factors, whereas we transferred equal numbers of Tet^H60+^ cells. Finally, we transplanted our cells into mice along with naive donor T cells to ensure that experimental cells were competed in a GVH environment.

We also explored the importance of TCF-1 itself in the initiation of the alloreactive CD8 response. TCF-1 is a multifunctional protein with different domains that play distinct roles in context-specific fashions (44). The amino terminus domain (absent in *p45^–/–^* cells) binds β-catenin, which is stabilized and translocated to the nucleus downstream of Wnt-pathway engagement. Canonically, the β-catenin/TCF-1 complex has been thought to activate transcription, whereas in the absence of β-catenin, Groucho family members bound to a domain adjacent to the β-catenin binding domain at a site present in TCF-1 can lead to transcriptional repression (45). Other domains (from the amino to carboxy terminus) include an intrinsically disordered domain, a histone deacetylase domain, and a high-mobility group DNA binding domain that may also associate with other transcription factors (44, 46, 47). Short TCF-1 isoforms that lack the β-catenin binding site can still bind Groucho family members and potentially repress transcription even when β-catenin is available in the nucleus.

We compared WT and *p45^–/–^* CD8 cells in several contexts. When transplanted into 129 mice, *p45^–/–^* CD8 cells were greatly outcompeted by WT CD8 cells in all tissues and at all times post-BMT, among both Tet^H60+^ and Tet^H60–^ cells. In contrast, *p45^–/–^* T cells were not disadvantaged in syngeneic transplants, which is driven by lymphopenia-induced proliferation (33) yet still relies on TCR engagement (32). One trivial explanation for the defective GVH response by *p45^–/–^* CD8 cells could be that the precursor frequencies of H60-reactive and miHA-reactive T cells are lower among them. That we saw similar Tet^H60+^ responses by *p45^–/–^* and WT CD8 cells with H60-immunization argues against that. These vaccination results also demonstrate that TCF-1 long isoforms are not essential for responses against H60 when it is presented transiently in contrast to when H60 is presented in a sustained fashion in the context of a GVH response.

Somewhat surprisingly, *p45^–/–^* and WT CD8 cells when transplanted separately mediated similar GVL. Moreover, the striking disadvantage when *p45^–/–^* cells were competed with WT cells was not evident in the numbers of WT and *p45^–/–^* CD8 cells recovered. Similarly, it was unexpected that WT and *p45^–/–^* CD8 would cause similar GVHD pathology in the B6→C3H.SW model, though there was less weight loss in the *p45^–/–^* recipients.

Our results need to be taken in the context of other studies of TCF-1. In LCMV and listeria models, the complete absence of TCF-1 (TCF-1^null^) only modestly impaired acute effector CD8 responses, whereas in contrast, TCF-1 was critical for T_CM_ formation and expansion upon rechallenge (48, 49). Only the p45, and not the p33 isoform, when expressed constitutively in *Tcf7*^–/–^ CD8 cells, restored T_CM_ formation and function (49). Consistent with this, *p45^–/–^* P14 TCR-transgenic CD8^+^ T cells mounted relatively normal acute responses against LCMV Armstrong but generate fewer CD62L^+^ progeny, which produced less IL-2 upon restimulation (50). In chronic viral infection, durable antiviral immunity is defective with TCF-1^null^ CD8 cells; however, to our knowledge, the roles of the short and long isoforms and the β-catenin–dependent functions of TCF-1 in generating and maintaining T_PEX_ have yet to be definitively clarified (12, 13, 15).

Most relevant to our work, Mammadli et al. recently reported that CD4 cells from *Tcf7*^fl/fl^ × CD4-cre mice (*Tcf7*^–/–^) which lack all *Tcf7* isoforms, failed to induce clinical GVHD in the B6→BALB/c model (51). In spleen and liver, there were fewer *Tcf7*^–/–^ T cells at day +7 without differences in proliferation or death. Tox expression was also similar in WT and *Tcf7*^–/–^ CD4 cells (51). Separately, the same group reported that *Tcf7*^–/–^ CD8 cells failed to induce clinical GVHD in the B6→BALB/c model but did mediate GVL (52).

Taken together, these prior studies and our results indicate that the β-catenin–independent functions of TCF-1, perhaps dominantly mediated by the short TCF-1 isoforms, are key for conferring GVHD potential on CD8^+^ T cells. Because GVL was intact with TCF-1^–/–^ CD8 cells, leukemia control in the minimal residual disease models studied by Harris (52) and our group are most likely to be T_EFF_ dependent, akin to the effectiveness of TCF-1^null^ CD8 cells in acute infection models.

Nonetheless, *p45^–/–^* CD8 cells were still disadvantaged in the competitive setting. It has been reported that TCF-1 is important for T cell responses to IL-7 and IL-15 (47), and this difference could have been amplified in the competitive setting. Alternatively, *p45^–/–^* cells may express less TCF-1 than do WT cells (37), and this could also have contributed. Definitively characterizing the roles of TCF-1 and its isoforms in GVHD initiation will require testing TCF-1^–/–^ CD8 cells created by the inducible deletion of TCF-1 after the peripheral CD8 compartment has been formed to *p45^–/–^* CD8 cells in the same GVHD models. Ideally, both gene-modified mice would be crossed to a TCR transgenic such that potential TCR repertoire differences can be definitively accounted for, as has been done in LCMV model systems. Finally, it will be important to determine the role of TCF-1 in maintaining intratissue GVHD responses, which will require its inducible deletion after GVHD has already been established.

## Methods

### Sex as a biological variable.

To control for potential contributions by T cell responses against male Y-chromosome encoded miHAs, only female recipients were used. This also enabled the use of both male and female BM and T cell donors.

### Mice.

B6.SJL-*Ptprc*^a^ (B6 CD45.1), B6.PL-*Thy1*^a^; (B6 CD90.1), 129S1/SvImJ (129), and C3H.SW mice were purchased from The Jackson Laboratory (JAX). B6 *Tcf7*^p45–/–^ mice (*p45^–/–^*), which lack the N-terminal β-catenin binding domain of TCF-1, were generated as described and obtained from JAX (36, 37). All mice were housed under specific pathogen-free conditions at the University of Pittsburgh in accordance with IACUC protocols.

### Cell purifications and sorting.

BM for transplant was obtained from pelvis, femur, and tibia bones of B6 CD45.1 donors and T cell depleted using a CD90.2 positive selection kit (StemCell Technologies). CD4 and CD8 cells were purified from donor spleens using CD4 and CD8 negative selection kits as per manufacturer’s protocol (Stem Cell Technologies). As indicated, cell sorting was performed using a FACS Aria (BD Biosciences).

### BMT.

Recipient mice were lethally irradiated with 1,000 cGy using a cesium-137 source 24 hours prior to transplant. In the B6→129 model, all mice received 5 × 10^6^ T cell–depleted BM cells, with 5 × 10^5^ CD4 cells and a total CD8^+^ T cell dose containing 2 × 10^6^ naive cells (CD44^–^CD62L^+^) unless otherwise specified. In the B6→C3H.SW model, treatment was the same except mice received 2 × 10^6^ CD8^+^ T cells from WT B6 CD90.1^+^ or *p45^–/–^* CD90.1^+^ mice. Cells were administered via lateral tail vein injection.

### Leukemia induction.

Murine blast-crisis chronic myeloid leukemia cells were generated as previously described (40). Briefly, BM from 5FU-treated 129 mice was spin infected with 2 retroviruses, one expressing the *bcr-abl* fusion gene linked by an internal ribosome entry site to a truncated nonsignaling human nerve growth factor receptor (hNGFR) reporter, and a second expressing the NUP98-HOXA9 with a GFP reporter. Transduced BM was administered to sublethally irradiated 129 primary hosts (600 cGy) for expansion. Premorbid mice were sacrificed and leukemic splenocytes were passaged into irradiated secondary 129 hosts, from which spleens were frozen and profiled for leukemia purity by quantitating the number of CD11b^–^hNGFR^+^GFP^+^ cells.

### Tissue processing.

Labeling of circulating intravascular cells was achieved by administration of anti-CD45.2-FITC antibody (3 μg/mouse, clone 104, BioLegend) via tail vein injection 3 minutes prior to animal sacrifice. Blood was obtained immediately prior to sacrifice by facial vein bleeds into heparinized tubes (0.5 mg/mL in PBS, Sigma), followed by red-cell lysis with ACK lysis buffer (Thermo Fisher Scientific). Mesenteric lymph nodes (mLN) and spleens were mechanically dissociated through a 70 μm cell strainer to obtain single-cell suspensions. Skin was enzymatically dissociated by incubating ears in collagenase IV for 2 hours at 37°C (1 mg/mL, Sigma) followed by trituration through an 18G needle. Livers were perfused with PBS immediately after sacrifice, excised, minced with scissors, and pressed through 100 μm cell strainers before digestion in collagenase IV (1 mg/mL) and DNase (2 μg/mL, Sigma) at 37°C for 40 minutes. Lymphocytes were then isolated by Optiprep gradient centrifugation (Sigma). Small intestines and colons were sectioned longitudinally and washed in HBSS. To separate intraepithelial lymphocytes (IEL) from lamina propria (LP), small intestines were preincubated in RPMI containing 5 mM EDTA, 1 mM DTT and 3% calf serum at 37°C for 30 minutes. Tissues were then shaken in RPMI with 2 mM EDTA to dissociate IEL, which were then purified by gradient centrifugation with HistoPaque (Sigma). Remaining small intestine tissue was incubated with Liberase TL (0.1 mg/mL, Sigma) and DNase (20 μg/mL) at 37°C for 20 minutes to release lymphocytes from the LP. Colon was processed in a similar fashion, but without separation of IEL from LP.

### GVHD histopathologic scoring.

Portions of liver, colon, terminal ileum, ear, and intrascapular skin were fixed in 10% formalin, paraffin embedded, sectioned, mounted and stained with H&E. Gastrointestinal and skin samples were scored by researchers blinded to experimental groups. Histologic scoring criteria were implemented as previously described (38). Skin GVHD scores presented herein are a sum of dermis, epidermis, inflammation, and follicle component scores (38).

### Flow cytometry and antibodies.

For surface antibody staining, single-cell suspensions from tissues were incubated at room temperature for 30 minutes in PBS with 2 mM EDTA and 3% calf serum with the following antibody conjugates: CD8-BUV395 (clone 53-6.7, BD Bioscience), CD4-BUV496 (GK1.5, BD Bioscience), CD19-BUV737 (1D3, BD Bioscience), CD229.1-BUV805 (30C7, BD Bioscience), CD44-BV480 (IM7, BD Bioscience), CD45.1-AF700 (A20, BioLegend), CD45.2-BV750 (104, BioLegend), CD90.1-BV650 (OX-7, BioLegend), CD90.2-BV785 (30-H12, BioLegend), CD62L-BV570 (MEL-14, BioLegend), PD-1-BV711 (29F.1A12, BioLegend), Tim-3-BV605 (RMT3-23, BioLegend), hNGFR-PE/Cy7 (ME20.4, BioLegend), CD39-PerCP-eFluor710 (24DMS1, Thermo Fisher Scientific), and H2-K^b^:H60 (LTFNYRNL)-BV421 tetramer (NIH Tetramer Core). Cells were then washed, fixed, and permeabilized and intracellular stains were performed using the FoxP3 intracellular staining kit according to protocol (Thermo Fisher Scientific). Intracellular stains included Tox-PE (TXRX10, Thermo Fisher Scientific), N-terminal TCF-1–AF647 (C63D9, Cell Signaling, used throughout this study unless otherwise specified), and unconjugated C-terminal TCF-1 (C46C7, Cell Signaling) with anti-rabbit-AF647 secondary (BioLegend Poly4064). All samples were analyzed on a Cytek Aurora 5L cytometer (Cytek) and analysis was performed using FlowJo software (BD Bioscience).

### H60 vaccination.

To acutely challenge against H60 antigen, splenocytes from B6 CD90.1 and *p45^–/–^* mice were mixed in a 1:1 ratio (1 × 10^6^ CD8^+^ CD44^–^CD62L^+^ T_N_ each) and delivered via lateral tail vein injection into B6 CD45.1 recipients. Immediately following, 50 μg of DEC-H60, an anti-DEC-205 antibody modified to express the immunogenic epitope of H60 (LTFNYRNL) (28) was coadministered with 50 μg of FGK-45 (BioXCell), an anti-CD40 agonist antibody, via the contralateral tail-vein.

### Statistics.

All quantitative data are presented as the mean ± SD. GraphPad Prism software was used for statistical analysis. Significance between groups was assessed using 1- or 2-sided Student’s *t* test, Mann-Whitney *U* test, or 1-way ANOVA as indicated. *P* values less than 0.05 were considered significant.

### Study approval.

All experiments with mice were approved by the University of Pittsburgh IACUC.

### Data availability.

All data points presented in graphs are available in the Supporting Data Values file.

## Author contributions

WS conceived of experiments, analyzed data, and wrote the paper. KQ conceived and executed experiments, analyzed data, and wrote the paper. FS conceived experiments, assisted in execution of experiments, and provided technical advice. SR, ERM, KCC, LGD, AMR, and WW assisted in the execution of experiments. DJ and JMM scored GVHD tissues.

## Supplementary Material

Supplemental data

Supporting data values

## Version 1 10/08/2025

Electronic publication

## Version 2 10/08/2025

symbol / font corrections
