# Supplementary material for: The role of TCF-1+CD8+ exhausted progenitors and TCF-1 in graft-versus-host responses
Source: JCI Insight. 2025 Oct 8;10(19):e181568. doi: 10.1172/jci.insight.181568 (PMC12507345; doi:10.1172/jci.insight.181568)
Supplement: Supplemental data [file jciinsight-10-181568-s270.pdf]

Supplemental Figure 1

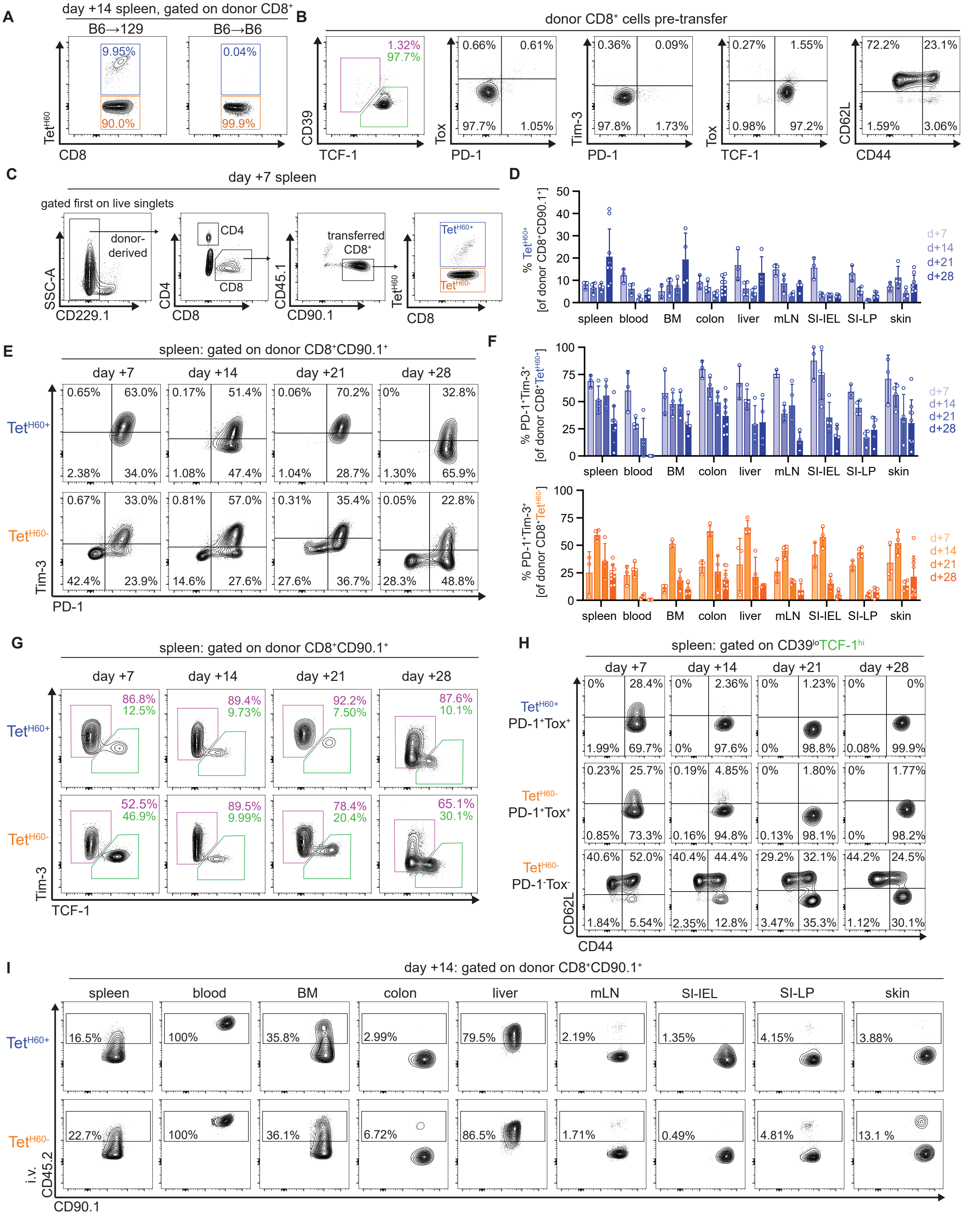

Supplemental Figure 1. Phenotypes and representative staining of T cells in B6→129 transplants. (**A**) Representative H-2K^{b}:H60 tetramer staining of donor B6 CD8^{+} cells 14-days post transplant in spleens of allogeneic 129 (H60^{+}) and syngeneic B6 (H60^{-}) recipients. (**B**) Immunophenotypes of donor B6 CD8^{+} T cells prior to transplant. (**C**) Gating strategy used to identify and classify donor CD8 cells according to H60-reactivity based on Tet^{H60} staining. (**D**) Percentages of Tet^{H60+} CD8 cells in tissues at each timepoint. (**E**) Representative staining for PD-1 and Tim-3 over time in spleen and (**F**) quantitated across tissues by H60-reactivity. (**G**) Representative staining of TCF-1 and Tim-3 on splenic donor CD8 cells. (**H**) Representative CD44 vs. CD62L staining in spleen among Tet^{H60+}PD1^{+}Tox^{+}, Tet^{H60-}PD-1^{+}Tox^{+} and Tet^{H60-}PD-1^{-}Tox^{-} populations. (**I**) Identification of intravascular and extravascular donor CD8^{+} cells by intravenous CD45.2 antibody labeling 3-minutes prior to animal sacrifice.

Supplemental Figure 2

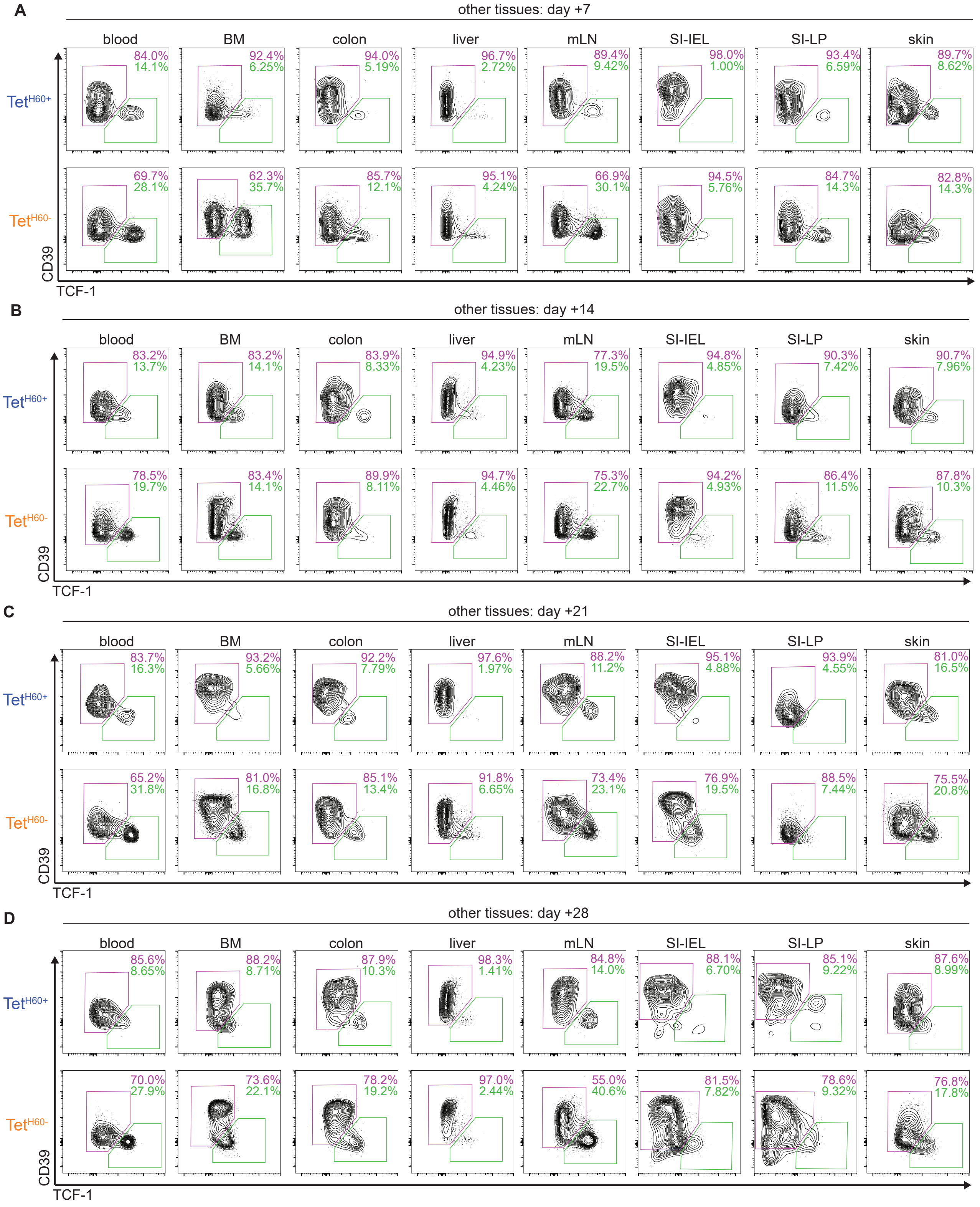

Supplemental Figure 2. Representative staining of TCF-1 vs CD39 in tissues on donor-derived CD8+ T cells at (**A**) day +7, (**B**) day +14, (**C**) day +21 and (**D**) day +28.

Supplemental Figure 3

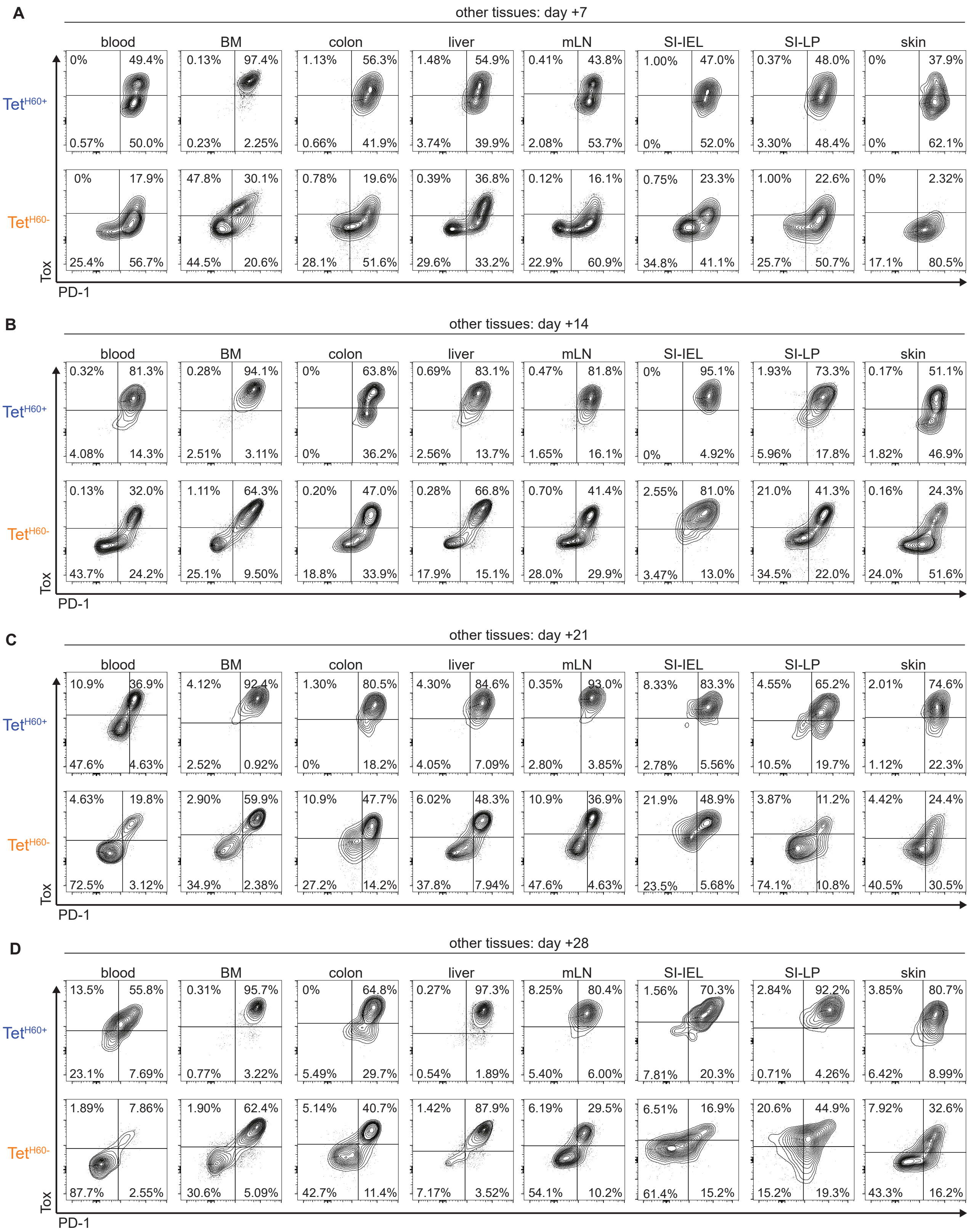

Supplemental Figure 3. Representative staining of PD-1 vs Tox on donor-derived $CD8^+$ T cells in tissues at (**A**) day +7, (**B**) day +14, (**C**) day +21, and (**D**) day +28.

Supplemental Figure 4

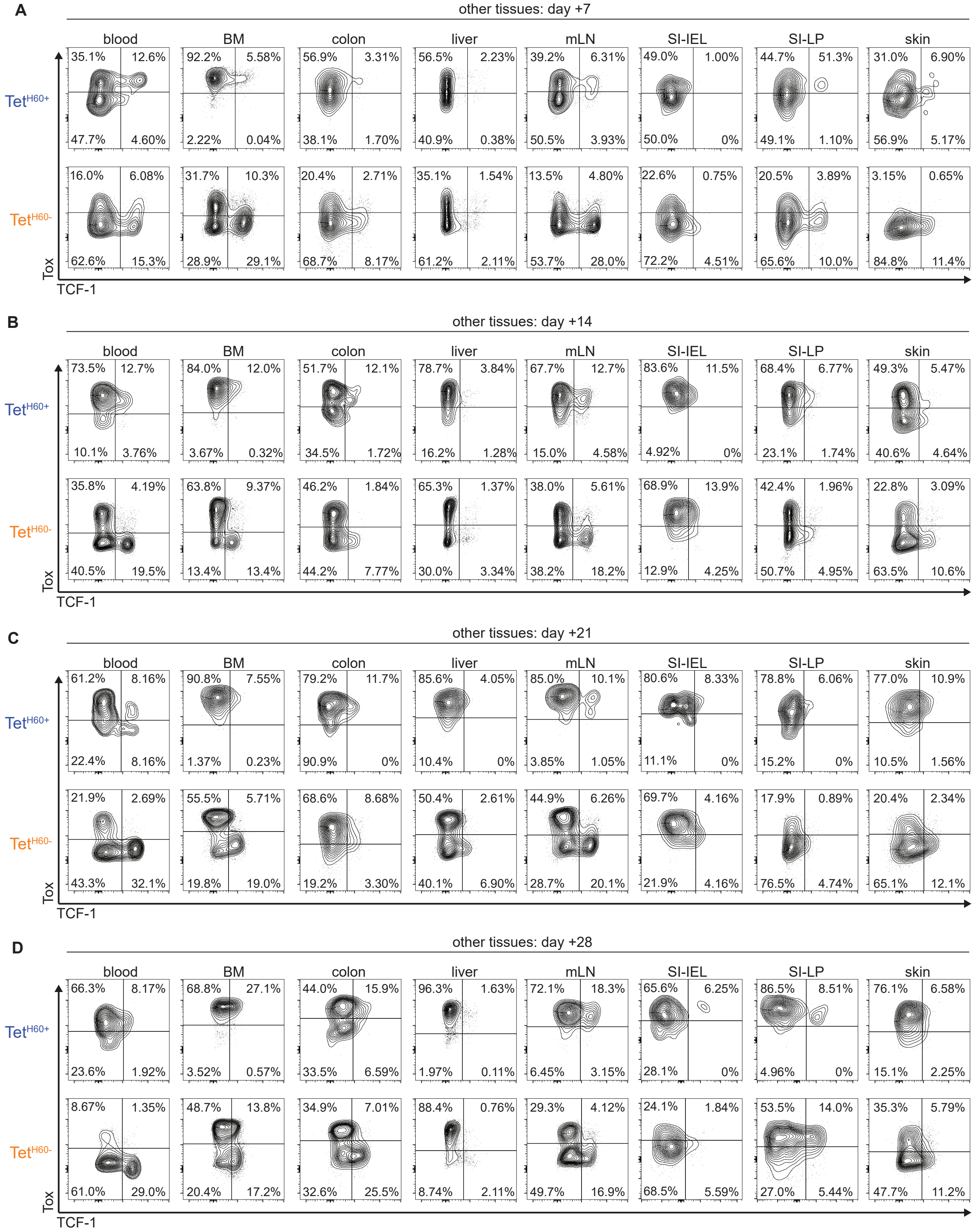

Supplemental Figure 4. Representative staining of TCF-1 vs Tox on donor-derived CD8$^+$ T cells in tissues at (**A**) day +7, (**B**) day +14, (**C**) day +21, and (**D**) day +28.
